# Exemplar Hospital Initiation Trial to Enhance Treatment Engagement (EXHIT ENTRE): protocol for CTN-0098 an open-label randomized comparative effectiveness trial of extended-release buprenorphine versus treatment as usual on post-hospital treatment engagement for hospitalized patients with opioid use disorder

**DOI:** 10.1186/s13722-024-00510-5

**Published:** 2024-12-02

**Authors:** Gavin Bart, Kelly S. Barth, Paulette Baukol, Eva Enns, Udi E. Ghitza, Jacklyn Harris, Eve Jelstrom, Jane M. Liebschutz, Kara M. Magane, Delia Voronca, Zoe M. Weinstein, P. Todd Korthuis

**Affiliations:** 1 Department of Medicine G-5, Hennepin Healthcare and University of Minnesota, 701 Park Avenue, Minneapolis, MN 55415 USA; 2 Department of Psychiatry & Behavioral Sciences, Medical University of South Carolina, 67 President Street, Charleston, SC 29425 USA; 3 Berman Center for Outcomes & Clinical Research, 701 Park Ave, Ste. PP7.700, Minneapolis, MN 55415 USA; 4 Division of Health Policy and Management, University of Minnesota School of Public Health, 420 Delaware St. SE, Minneapolis, MN 55408 USA; 5 National Institute on Drug Abuse (NIDA) Center for the Clinical Trials Network (CCTN), Bethesda, MD 20892 USA; 6 The Emmes Company, LLC, 401 N. Washington St. #700, Rockville, MD 20850 USA; 7 Division of General Internal Medicine, Center for Research on Healthcare, University of Pittsburgh, UPMC, Pittsburgh, PA 15213 USA; 8 Boston University School of Public Health, 801 Massachusetts Ave, Suite 431, Boston, MA 02118 USA; 9 Regeneron Pharmaceuticals, Inc, 777 Old Saw Mill River Rd, Tarrytown, NY 10591-6707 USA; 10 Grayken Center for Addiction, Clinical Addiction Research and Education Unit, Section of General Internal Medicine, Department of Medicine, Boston Medical Center, Boston University Chobanian & Avedisian School of Medicine, 801 Massachusetts Ave. 2nd Floor, Boston, MA 02118 USA; 11 Department of Medicine, Addiction Medicine Section, Oregon Health & Science University, 3181 SW Sam Jackson Park Rd, Portland, OR 97239-3098 USA

**Keywords:** Opioid use disorder, Medications for opioid use disorder, Comparative effectiveness, Protocol, Hospital

## Abstract

**Background:**

Hospitalizations involving opioid use disorder (OUD) are increasing. Addiction consultation services (ACS) initiate medications for opioid use disorder (MOUD) in hospital settings and arrange post-hospital follow-up for ongoing MOUD care. Engagement in MOUD following hospital discharge is hampered by challenges in timely access to MOUD. This protocol describes an open-label randomized comparative effectiveness trial comparing ACS treatment as usual (TAU) to a single injection of a 28-day formulation extended-release buprenorphine (XR-BUP) on MOUD engagement 34-days following hospital discharge.

**Methods:**

Six U.S. hospitals with ACS capable of prescribing all MOUD (i.e., methadone, buprenorphine, and extended-release naltrexone) recruit and randomize hospitalized patients with OUD who have not been on MOUD in the fourteen days prior to hospitalization. TAU may consist of any MOUD other than XR-BUP. Participants randomized to XR-BUP may receive any MOUD throughout their hospital stay and receive a 28-day XR-BUP injection within 72-hours of anticipated hospital discharge. There is no intervention beyond hospital stay. Participants are followed 34-, 90-, and 180-days following hospital discharge. The primary outcome is engagement in any MOUD 34-days following hospital discharge, which we hypothesize will be greater in the XR-BUP group. Randomizing 342 participants (171 per arm) provides 90% power to detect difference in the primary outcome between groups with an odds ratio of 2.1. Safety, secondary, and exploratory outcomes include: adverse events, MOUD engagement on days 90 and 180, opioid positive urine drug tests, self-reported drug use, hospital readmissions and emergency department visits, use of non-opioid drugs, fatal and non-fatal opioid overdose, all-cause mortality, quality of life, and cost-effectiveness. Data are analyzed by intention-to-treat, with pre-planned per-protocol and other secondary analyses that examine gender as an effect modifier, differences between groups, and impact of missingness.

**Discussion:**

Engagement in MOUD care following hospitalization in individuals with OUD is low. This randomized comparative effectiveness trial can inform hospital ACS in medication selection to improve MOUD engagement 34-days following hospital discharge.

**Trial registration:**

NCT04345718.

## Background

Hospitalizations for opioid use disorder (OUD) have increased substantially over the past decade. Between 2016 and 2018, there were nearly 1 million hospitalizations related to opioid use disorder (OUD) annually in the United States [1]. While skin and soft tissue infections comprise a large segment of these hospitalizations, patients with OUD are also hospitalized for a wide spectrum of other conditions with major depression, acute kidney injury, and exacerbations of obstructive lung disease among the most common [1–4]. Few people are offered effective interventions for OUD during hospitalization, which may contribute to the 8% all-cause mortality rate observed in these patients in the year following hospital discharge [5, 6]. 

Medications for opioid use disorder (MOUD), especially methadone and buprenorphine, reduce all-cause mortality by more than 50%, reduce risk of infection with HIV and hepatitis C, and improve several social determinants of health [7–10]. Hospitalization is an opportune time to engage patients in MOUD [11, 12]. Failure to do so not only increases post-hospitalization morbidity and mortality but can complicate the course of care while the patient is still hospitalized and increase discharges before medically advised, hospital readmission, and incomplete courses of treatment (e.g., insufficient antibiosis) [13–16]. Increasingly, hospitals have adopted addiction medicine consultation services (ACS) to address MOUD initiation and other aspects of care for people who use drugs (PWUD) [17]. These services improve linkage to ongoing MOUD care post-hospitalization and reduce hospital readmission rates compared to patients not seen by ACS [18–20]. Timely linkage to ongoing MOUD following hospital discharge has been a challenge, however [21]. In one study, an average of 16 days was needed for patients to access ambulatory addiction services [22]. Patients discharged on sublingual buprenorphine often receive less than one-week of medication upon discharge, creating a risky gap where patients may face withdrawal, loss of tolerance, increased overdose risk, return to opioid use, and loss to follow-up. Rapid access to outpatient MOUD within one- or two-days following discharge decreases this risk but is challenging to achieve [23]. The advent of extended-release injectable buprenorphine (XR-BUP) in 7- and 28-day formulations may improve ongoing engagement in MOUD in community settings [24, 25] but little is known about its impact on facilitating transitions of care from hospital to community care.

This open-label randomized trial in hospitalized patients with OUD who have not been on MOUD in the 14 days prior to admission, tests the comparative effectiveness of a 28-day formulation of XR-BUP administered within 72 h of anticipated hospital discharge versus ACS treatment as usual (TAU, consisting of either methadone or sublingual buprenorphine) on MOUD treatment engagement 34-days following hospital discharge. We hypothesize that XR-BUP, which offers more time for successful linkage than TAU, will result in a greater proportion of patients engaged in MOUD 34-days post-hospitalization. This outcome is adapted from the Health Effectiveness Data and Information Set (HEDIS) Initiation and Engagement of Substance Use Disorder Treatment (IET) measure used by more than 90% of health plans and specified by the National Committee on Quality Assurance (NCQA) [26, 27]. The measure, while imperfect, allows evaluation of treatment engagement up to 34 days following hospital discharge and has been validated in studies that find associations between meeting the measure and improved patient outcomes [20, 28]. While HEDIS defines engagement as two or more alcohol or other drug services within 34 days of initiation of alcohol and other drug dependence treatment, here we modify the measure for OUD treatment, using treatment with a prescribed MOUD on day 34 as the primary outcome since exposure to MOUD in itself represents engagement. This adaptation has face validity in Medicaid data [29]. It should be noted that since this study was designed, HEDIS has developed a pharmacotherapy for opioid use disorder measure defined as the percentage of patients continuing MOUD for 180 days.

## Methods

### Study objectives and design

Funded by the NIH HEAL Initiative^®^ and conducted through a cooperative agreement in the National Institute on Drug Abuse (NIDA) National Drug Abuse Treatment Clinical Trials Network (CTN), CTN-0098A Exemplar Hospital Initiation Trial to Enhance Treatment Engagement (EXHIT ENTRE) is a multi-site open-label randomized comparative effectiveness trial of XR-BUP versus TAU for hospitalized patients with untreated OUD prior to hospital admission who are willing to initiate MOUD while hospitalized. The primary objective of this study compares MOUD engagement 34-days following hospital discharge in hospitalized patients randomized to ACS TAU versus a single injection of a 28-day formulation of XR-BUP prior to discharge. Secondary objectives compare safety, engagement in MOUD care at 90- and 180-days post discharge, drug use, hospital readmissions, and emergency room visits among participants randomized to TAU versus XR-BUP.

**Fig. 1:**
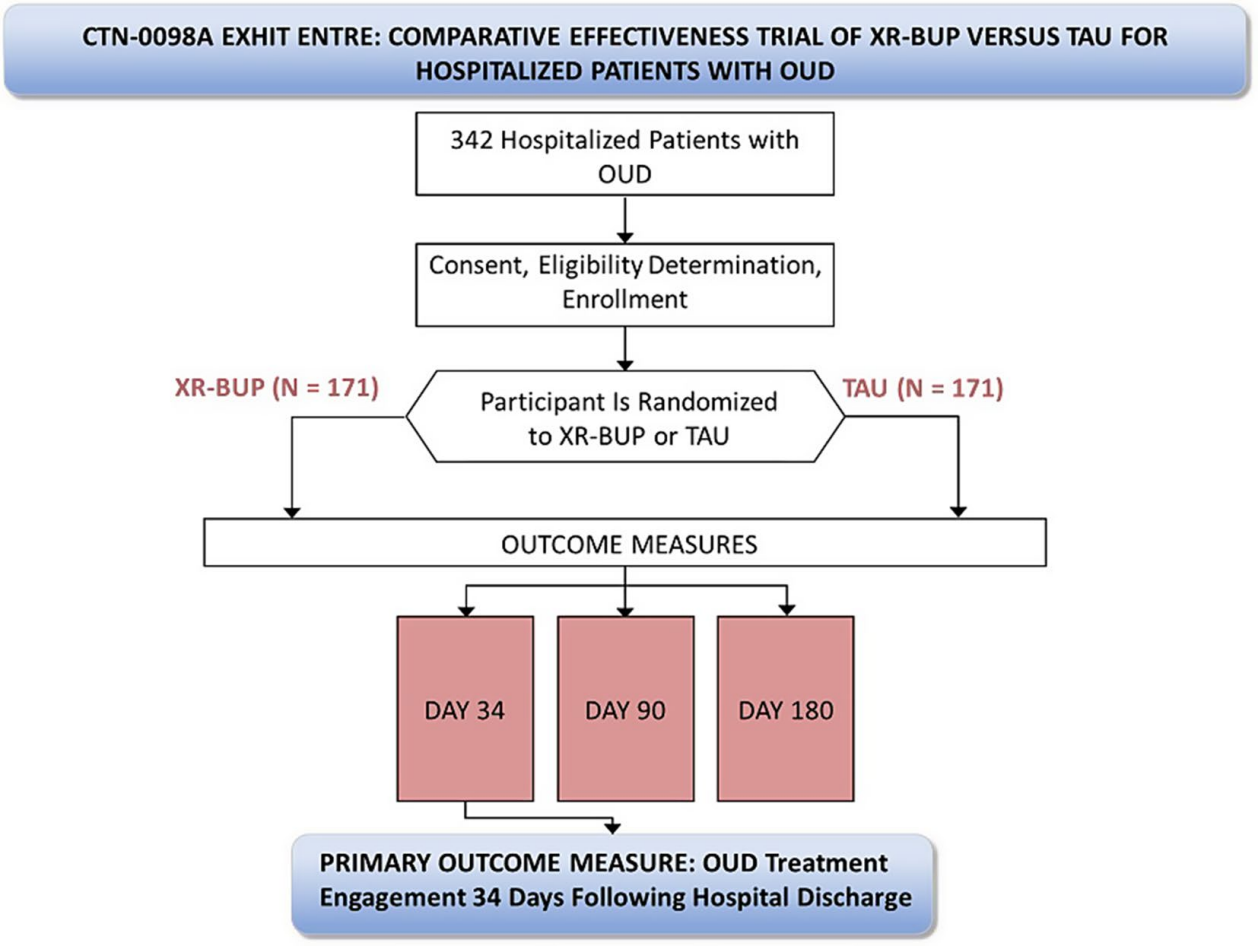
Study Schema

Advarra, a single independent commercial institutional review board (IRB), approved the study (Advarra Pro00047336), with all sites ceding to this single IRB. The study was also reviewed by an independent Protocol Review Board and subsequently a Data and Safety Monitoring Board (DSMB) appointed by the NIDA Center for the Clinical Trials Network (NIDA CCTN). The sponsor does not make decisions regarding publication of this or other study-related manuscripts (Fig. 1).

### Study setting and site selection

Solicitation for hospitals to participate in the study are distributed through the NIDA CTN. To be eligible for participation, hospitals must have established ACS with the capacity to initiate all FDA-approved MOUD. Five hospitals with established ACS were recruited initially, with a sixth hospital added after study initiation to improve recruitment (for sites see https://clinicaltrials.gov/study/NCT04345718).

**Participating sites must**:

1. Have an existing ACS with experience initiating MOUD prior to hospital discharge.
2. Have a buprenorphine waivered provider “champion” on the ACS who can commit the time required to oversee medical aspects of the study, perform medical assessments, confirm participant eligibility, order study medications, and evaluate and respond to adverse events that may occur during the study period (study protocol was developed prior to the 2022 X-waiver elimination).
3. Have at least one other buprenorphine waivered provider able to perform medical aspects of this study.
4. Have the staff required to recruit and retain participants.
5. Have the staff required to collect research data (including biological specimens such as blood and urine).
6. Have access to local laboratory services to process screening and monitoring specimens (e.g., blood and urine).
7. Be able to manage (store, dispense, and dispose) study medication including having an XR-BUP Risk Evaluation and Mitigation Strategy (REMS) participating pharmacy, if required.

### Study participants

**Eligible participants must be**:

1. Hospitalized.
2. At least 18 years of age.
  - Children are not included because addiction health services for them present different challenges and their access to methadone treatment is greatly restricted thus preventing adequate outcomes analysis.
3. Meet DSM-5 criteria for moderate or severe OUD.
4. Willing to initiate MOUD, including buprenorphine.
5. Able to speak English sufficiently to understand the study procedures and provide written informed consent to participate in the study.

**Participants are excluded from participation if**:

1. Anticipated hospital length of stay less than 24-hours as determined by the ACS.
2. Affected by a serious medical, psychiatric, or substance use disorder that, in the opinion of the study physician, would make it unsafe to participate in the study or may prevent collection of study data. This may include:

- Disabling terminal diagnosis for which discharge from hospital is not anticipated or for which hospice care is being sought.
- Severe alcohol or benzodiazepine use disorder that is anticipated to require complex medical detoxification which cannot be completed prior to randomization.

3. Taking a long-acting opioid other than buprenorphine (e.g., methadone, extended-release oxycodone, extended-release morphine) for each of the three days immediately preceding randomization.
4. Liver enzyme tests (Aspartate Aminotransferase (AST), Alanine Aminotransferase (ALT)) more than 3 times upper limit of normal.
5. Currently pregnant or breastfeeding.
6. Known allergy to buprenorphine or components of FluidCrystal delivery system.
7. Receipt of MOUD in the 14 days prior to hospitalization as maintenance treatment; however, patients may have received MOUD for withdrawal management during or prior to hospitalization at the time of enrollment.
8. Are currently in jail, prison or other overnight facility as required by court of law and/or is considered a prisoner under local law or is under current terms of civil commitment or guardianship.
9. Previously randomized as a participant in the study – individuals may only be enrolled and randomized once.

### Participant recruitment and consent

Recruitment procedures vary by site, but potential participants are drawn from each hospital’s pool of inpatients who have been identified by the ACS as having a possible OUD. Clinical or research staff approach these patients during their index admission to provide information about the study. Individuals providing verbal consent are prescreened under a HIPAA Waiver of Authorization by reviewing the electronic medical record to further assess inclusion/exclusion criteria. If a person meets initial eligibility criteria during the prescreening process, they give written informed consent prior to completing screening and baseline procedures. The informed consent process includes a quiz testing comprehension of study activities, alternatives, and potential risks and benefits, which may be repeated until answered correctly. Participants can opt to provide additional consent for blood collection and biobanking for potential future addiction- and MOUD- related genetic studies. Baseline data collection is completed prior to randomization.

### Randomization

Approximately 342 eligible participants are randomized in a 1:1 ratio to open-label XR-BUP or TAU (e.g., methadone, sublingual buprenorphine, extended-release naltrexone). The randomization process is performed by computer by a central data and statistics center (DSC). A permuted block randomization procedure with random block sizes is used. The DSC statistician generates the randomization schedule using blocks of varying sizes within strata (hospitals) to ensure lack of predictability along with relative equality of assignment across treatment groups. The randomization details such as block size are not conveyed to staff or participants. The DSC statistician reviews randomization data on a regular basis to ensure that the scheme is being implemented according to plan. A randomization slot, once used, is not re-allocated.

### Intervention

This study randomly assigns participants to ACS TAU or a single injection of 28-day formulation XR-BUP administered within 72 h of anticipated hospital discharge. This study is open-label and not blinded. There are no study interventions that extend beyond the index hospitalization or to facilitate care post-hospitalization or MOUD adherence beyond the standard care provided by the ACS. The ACS may initiate MOUD at any point during hospitalization. TAU may consist of any FDA-approved MOUD (other than XR-BUP) with the dose and amount of MOUD supplied upon hospital discharge determined by the ACS. For methadone, federal regulation prevents more than three days of dispensing outside of an opioid treatment program, whereas up to 30-days of buprenorphine may be provided upon hospital release. Although participants randomized to XR-BUP do not receive their XR-BUP injection until they are within 72-hours of anticipated hospital discharge, the ACS may offer other MOUD during their hospitalization per local standard of care, which reduces potential imbalance between arms in approaches to MOUD during recruitment and screening. The rationale for not providing XR-BUP until just prior to hospital discharge is to account for variable length of hospital admissions and prevent some participants receiving an injection early in a prolonged hospitalization where the effect of XR-BUP may wear off during hospitalization or shortly thereafter.

MOUD initiation strategies are determined by the ACS and doses titrated per their standard of care. Participants randomized to XR-BUP must tolerate at least one dose of sublingual buprenorphine prior to injection. As XR-BUP is available in three doses, participants can receive a dose commensurate with their current (if on a stable dose) or anticipated stable dose of sublingual buprenorphine. Available doses of XR-BUP (equivalent sublingual dose range) are: 64 mg (8–10 mg), 96 mg (12–16 mg), and 128 mg (18–24 mg). Because the lowest XR-BUP dose approximates 8 mg sublingually, participants are required to have an anticipated maintenance dose of at least 8 mg sublingually. Access to XR-BUP (CAM-2038, Braeburn, Inc.) is via an Investigational New Drug Application as it was not commercially available at time of study initiation, although it has since become available. The National Institute on Drug Abuse negotiated with Braeburn Inc. to procure donated XR-BUP for this study, the lead investigators were not part of this process.

Participant follow-up for outcome assessments occur 34-, 90-, and 180-days following discharge from the index hospitalization. Participants receive $50 for baseline assessment and enrollment and $50, $75, and $100, respectively for the three follow-up visits.

### Assessments

Study assessment and the schedule of study activities are presented in the Table. Where possible, assessments include validated tools that are used widely throughout other NIDA CTN studies. For example, the Substance Abuse and Addiction Collection of the PhenX Toolkit [30] is used at baseline to assess demographics (age, ethnicity, sex, race, educational attainment, employment status and marital status), BMI, Human Immunodeficiency Virus (HIV) Risk & Status, and substance use measures including age of onset, past 30-day quantity and frequency, and lifetime use for alcohol, tobacco and other substances.

While the Timeline Followback (TLFB) [31, 32] is used at each study visit to assess past month drug and alcohol use, we have adapted an additional TLFB that asks about daily use of MOUD in the specified timeframe for the 34, 90, and 180-day follow-up visits.

Tests for viral hepatitis (hepatitis B surface antigen and antibody, hepatitis C antibody with reflex PCR, if positive) and HIV with reflex viral load, if positive, are collected at baseline unless results from the 90-days prior to admission can be abstracted from the medical record.

Urine drug screens (UDS) are collected at screening/baseline and at each follow-up visit to assess secondary outcomes, unless the follow-up visit is fully remote. All urine specimens are collected using FDA-approved one-step temperature-controlled urine drug test cups testing for the presence of: opiates, oxycodone, barbiturates, benzodiazepines, cocaine, amphetamines, methamphetamines, marijuana, methadone, buprenorphine, phencyclidine (PCP), fentanyl, and ecstasy (MDMA). Results of UDS are not shared with participant clinical care givers.

Medical comorbidity is abstracted from the medical record problem list and/or discharge diagnoses as is the Comorbidity Severity Index (i.e., CMS-HCC Risk Adjustment [33]).

Pre- and post- XR-BUP injection evaluation for precipitated opioid withdrawal uses the Clinical Opioid Withdrawal Scale (COWS) [34] with a 5-point increase indicating precipitated withdrawal. An independent data and safety monitoring board (DSMB) convened by NIDA will have access to safety reports and will meet at least annually. The DSMB may receive aggregate data (blinded) as well as by randomization group (unblinded). The DSMB may recommend protocol modifications or even early study termination if either study arm has a clinically important excess of serious adverse events.

The majority of collected data are directly entered without personal identifiers into a secure electronic case report form (eCRF) system maintained by the DSC (Advantage eClinical). Data abstracted from the electronic health record or requiring a paper source CRF are transcribed from paper CRF to Advantage eClinical by study staff. Following study completion and publication of the primary outcome paper, as per NIH and HEAL Initiative policy, study data will be available through the NIDA data repository (Table 1).

**Table 1:** Table of assessments

	Prescreening	Screening/ Baseline^a^	Randomization	TAU/XR-BUP Initiation	Date of Discharge	Follow-Up			As Needed
DAY					0	34	90	180	
**General measures**									
Prescreen Approach Log	X								
Verbal Consent	X								
Prescreening Form	X								
Written Informed Consent and Quiz		X							
Inclusion/Exclusion		X							
Locator Form		X				X	X		
PhenX [30] Core Tier 1 Forms		X							
Demographics Forms		X							
Treatment Satisfaction Survey								X	
Treatment Initiation and Non-Initiation				X					
Hospitalization MOUD					X				
Marijuana Use Assessment		X							
COVID-19 Impact Assessment		X				X	X	X	
Timeline Followback (TLFB) (Medications)						X	X	X	
Study Completion Form								X	
**Measure of Primary and Secondary Outcomes**									
Engagement in MOUD Post Hospital Discharge						X	X	X	
Hospitalization and ED Utilization (Related or Not to OUD)						X	X	X	
Urine Drug Screen		X				X	X	X	
Timeline Followback (TLFB) (Drug/Alcohol) [31, 32]		X				X	X	X	
**Clinical and safety assessments**									
Medical and Psychiatric History		X							
Physical Examination		X							
Injection Site Examination^b^				X		X			
Injection Site Reaction Form^c^				X		X			X
Hospitalization Diagnoses					X				
Medical Comorbidity [33]					X				
Vital Signs		X							
DSM-5 Checklist [35]		X							
Mental Health Follow-Up Assessment									X
Adverse Events, Including Serious Adverse Events			X^d^	X	X	X	X	X^e^	
Prior and Concomitant Medications		X	X	X	X				
Clinical Opioid Withdrawal Scale^b; f^ [34]				X					
**Clinical laboratory assessments**									
Pregnancy and Birth Control Assessment^g^		X							
Confirmed Pregnancy and Outcome									X
Liver Transaminases (AST and ALT)^h^		X							
Hepatitis B, Hepatitis C, HIV Antibody^h^		X	X						
Genetic Sampling^h^		X	X			X	X	X	
Family Origin		X							
**Exploratory measures**									
Healthcare and Services Utilization [36]						X	X	X	
Pain Assessment [37]		X				X	X	X	
Depression (PHQ-9) [38]		X				X	X	X	
Post-Traumatic Stress Disorder (PC-PTSD-5) [39]		X				X	X	X	
Quality of Life (WHOQOL-BREF) [40]		X						X	
Non-Fatal Opioid Overdose		X				X	X	X	
Fatal Opioid Overdose (collected on SAE form)			X^d^	X	X	X	X	X	
Addiction Severity Index-Lite (ASI-Lite) Drug and Alcohol Use [41]		X						X	
Post-Hospitalization Medical Appointment Follow-Up						X	X		
Antibiotic Adherence for OUD-Related Infections^i^						X	X		
Hospital Length of Stay					X	X	X	X	
Subsequent XR-BUP Injections (XR-BUP group only)						X	X	X	
Receipt of Other MOUD						X	X	X	
**Administrative forms**									
Protocol Deviations									X
Missed Visit and Visit Documentation Form		X		X		X	X	X	

Notes: ^a^Can be completed at different time points; ^b^For XR-BUP only; ^c^Completed only if reaction noted upon injection site examination; ^d^AEs/SAEs collected only after the participant is randomized; ^e^At 180-days, only SAEs are collected; ^f^COWS is assessed twice (before and two-hours after XR-BUP initiation) for each participant randomized to XR-BUP group only; ^g^Completed a second time before treatment initiation if there are more than 7 days between screening/baseline and treatment initiation; ^h^Collected only once during these visits and not at each indicated time point; ^i^Completed only by participants discharged from the hospital on antibiotics for drug-related skin and soft-tissue infections

### Outcomes

The primary outcome measure is the proportion of participants engaged in MOUD care on the 34th day following hospital discharge. Engagement in MOUD care is defined as verifiable coverage with a prescribed MOUD on that 34th day regardless of the source of prescribed MOUD coverage (e.g., opioid treatment program, primary care, jail, etc.). Participants give appropriate permission for release of such data during informed consent procedures and/or during study follow-up visits. Other objective means of MOUD verification, such as bringing a prescription bottle with participant name and dates covered by the prescription can, also be used for primary outcome.

### Data for primary outcome

MOUD engagement on the 34th day post hospital discharge is collected by self-report verified with an objective data source (e.g., pill bottle, prescription record, provider confirmation upon release of information, etc.) or, in the absence of self-report, an objective data source is used for the primary outcome (e.g., electronic medical records, provider confirmation upon release of information, etc.).

**Secondary outcomes include**:

1. Proportion of participants that experience any treatment emergent AEs and SAEs (those developed after initiation of treatment) following hospital discharge.
2. Proportion of participants engaged with MOUD 90- and 180-days following hospital discharge.
3. Proportion of participants with positive urine drug test for opiates, fentanyl, and oxycodone 34-, and 90-, and 180-days following hospital discharge.
4. Proportion of participants with self-reported non-prescribed opioid use 34-, and 90-, and 180-days following hospital discharge.
5. Proportion of participants with self-reported hospital readmissions at the 34- and 90-day visits.
6. Proportion of participants with self-reported ED visits at the 34- and 90-day visits.

**Exploratory outcomes include**:

1. Proportion of participants with positive urine drug test for illicit drugs 34-, and 90-, and 180-days following hospital discharge.
2. Proportion of participants with self-reported non-prescribed drug use 34-, and 90-, and 180-days following hospital discharge.
3. Change in ASI-Lite measures from baseline to 180 days.
4. Proportion of participants with self-reported medical follow-up at the 34- and 90-day visits.
5. Proportion of participants adherent to antibiotic for OUD-related infections (e.g., endocarditis or skin-soft tissue infections) (when applicable).
6. Self-reported alcohol use 34-, and 90-, and 180-days following hospital discharge.
7. Hospital length of stay (for both the index hospitalization and readmissions).
8. Self-reported 180-day hospital readmission rates.
9. Self-reported 180-day ED visit rates.
10. All-cause mortality rates at 30-, 90- and 180-days following hospital discharge.
11. Comparative cost-effectiveness of XR-BUP and other MOUD.
12. Non-fatal opioid overdose rates (discharge to 34-day visit and since last study visit at the 90- and 180-day visits).
13. Fatal opioid overdose rates up to the 180-day visit.
14. Satisfaction with MOUD treatment 180 days following hospital discharge.
15. Self-reported 34- and 90-day hospital readmission rates related to OUD.
16. Self-reported 34- and 90-day ED visits related to OUD.
17. Quality of life.
18. Receipt of subsequent XR-BUP injections (among the XR-BUP group only).
19. Receipt of other MOUD treatments during follow-up (e.g., XR-BUP in the control group, methadone or SL-BUP in the XR-BUP group).

### Sample size

342 participants will be randomized in a 1:1 ratio to XR-BUP or TAU. The power calculation assumes the 34-day engagement rate is 0.39 for TAU [22] and 0.60 for XR-BUP and that 15% of the primary outcome values will be missing and treated as “not engaged” for the primary analysis. In that scenario, 85% of patients will have the assumed rate (0.39 and 0.60 for TAU and XR-BUP, respectively) and 15% of patients in both groups will have a rate of 0.0. As such, the new engagement rates will be 0.3315 for TAU and 0.51 for XR-BUP.

A Cochran-Mantel-Haenszel test with continuity correction based on odds ratios (OR) of 2.1 assuming different engagement rates by site (6 sites with 34-day engagement rates of 0.29 up to 0.37 for TAU and 0.46 up to 0.55 for XR-BUP group) suggests that a total of 342 participants (171 participants in each group) need to be enrolled to have 90% power to detect a difference in the 34-day engagement rates between TAU and XR-BUP (two-sided test, alpha [false positive rate] = 0.05). Following a blinded interim analysis, the DSMB may recommend the protocol be amended to increase the number of subjects enrolled into the study.

### Outcomes analyses

The primary analysis compares the two groups (TAU and XR-BUP) for the primary outcome (engagement in MOUD care on the 34th day following hospital discharge) using the Cochran-Mantel-Haenszel test stratified by hospital under the principle of intention-to-treat (ITT), i.e., participants are analyzed in the group to which they were randomized irrespective of whether they received their assigned treatment. An additional analysis may be performed using a per-protocol analysis population (i.e., according to the actual treatment received).

Supplementary analyses of the primary outcome will be performed as follows: (a) consider gender as an effect modifier, (b) add adjusters for possible differences between groups, and (c) adjust for missing outcome data. Logistic regression is used for (a) and (b) whereas analyses adjusting for missing outcome data will use inverse probability weighting (IPW).

Analyses of secondary outcomes, broadly, will have a form similar to analysis of the primary outcome, or outcomes measured at multiple time-points (e.g., engagement in OUD care at 34-, 90-, and 180-days) will use a mixed-effect analysis with a person-specific random effect to capture correlation of a person’s multiple time-points. The particular form of analysis, e.g., logistic regression vs. normal-errors regression, will depend on the outcome, and outcomes on continuous or effectively continuous scales may be transformed before analysis (e.g., by taking the logarithm) to allow use of analyses that assume normally distributed errors.

Analytic approaches for exploratory outcomes are similar to those described for the primary and secondary outcomes. In addition, we will explore moderators and mediators of MOUD engagement and other outcomes such as hospital length of stay and antibiotic completion rates (where appropriate). Potential moderators and mediators include hospital length of stay, post-hospital discharge medical follow-up, quality of life, depression, post-traumatic stress syndrome, pain, and treatment satisfaction.

Cost-effectiveness analysis will be conducted from a health sector perspective over the 6-month trial participation time horizon as well as over a remaining lifetime time horizon (by extrapolating from trial outcomes). Costs will include the healthcare costs associated with the initial OUD management pre-discharge (either a single dose of XR-BUP or TAU) and post-discharge healthcare utilization (ED visits and hospitalizations) and OUD care in the first six-months. Post-discharge costs will be estimated by multiplying self-reported healthcare utilization by the relevant Centers for Medicare and Medicaid Services (CMS) reimbursement rates [42]. 

The effectiveness of the interventions will be assessed in terms of quality-adjusted life-years (QALYs), which reflect both length and quality of life. We will estimate the average QALYs accrued under each intervention by calculating the amount of time participants spend in different OUD states (based on reported ED use, hospitalizations, and OUD care in the first 6 months) multiplied by the health-related utility of that state estimated from published data [10, 43]. Mortality is accounted for by assigning death a utility value of zero.

For the lifetime time horizon analysis, costs and QALYs after 6 months will be estimated by extrapolating from trial outcomes using estimated OUD outcomes from the published literature, economic analyses of MOUD treatments, and OUD modeling studies. We will conduct sensitivity analyses to assess the sensitivity of our cost-effectiveness conclusions on any extrapolation assumptions.

Once costs and QALYs have been assessed for XR-BUP and TAU, we will compare the incremental costs and benefits of XR-BUP. If XR-BUP yields a health benefit (increase in QALYs) and is less expensive than TAU, we will calculate the extent of this cost-savings to the healthcare sector. If XR-BUP is more expensive, but also more beneficial, than TAU, we will calculate the incremental cost-effectiveness ratio (ICER) of XR-BUP, which is the additional cost of each additional QALY gained by using XR-BUP for hospital-initiated MOUD instead of TAU. Cost-effectiveness conclusions will depend on the value of the ICER. In the US, interventions with an ICER less than $100,000 per QALY gained are considered cost-effective, between $100,000 - $150,000 per QALY gained are considered marginally cost-effective, and over $150,000 per QALY gained is considered not cost-effective.

### Trial progress

The study was approved by a single IRB in 2021 and recruitment is ongoing with more than 265 participants recruited by July 2024. Recruitment in hospitals was challenging during peak COVID-19 emergencies and supply chain disruptions led to a 6-week interruption in access to XR-BUP in 2022. The protocol is in its fifth version, with minor corrections to the previous versions.

## Discussion

This multi-site randomized open-label trial conducted in hospitalized patients with OUD and no MOUD in the 14 days prior to hospital admission evaluates the comparative effectiveness of addiction consultation service treatment as usual care versus a single pre-discharge injection of a 28-day formulation of XR-BUP on post-hospital day 34 engagement in MOUD care. As hospitalizations associated with OUD rise and hospitals begin to address this through in-hospital initiation of MOUD and post hospital transition to ongoing MOUD, this study will help inform best practices for improving treatment engagement following discharge.

The transition from hospital to ongoing community care post hospitalization is challenged by timely access to outpatient MOUD treatment [21]. We hypothesize that XR-BUP will provide stable MOUD coverage over 28 days, allowing more time to transition into community care and will thus result in more MOUD engagement 34-days after hospital discharge than TAU, which may require more rapid access to care than is typically available in the community. We acknowledge the potential for the opposite to be true, that the earlier engagement necessitated by TAU improves ongoing MOUD compared to XR-BUP, which may create too much temporal distance between hospitalization and engagement in outpatient care despite pharmacologic coverage with MOUD.

There are some limitations to this study design. All sites must have a functioning ACS; thus, findings may not generalize to hospitals that address OUD through other means. Further, we are not simply comparing sublingual buprenorphine to XR-BUP, rather treatment as usual can include methadone, sublingual buprenorphine, or XR-naltrexone, reflective of real-world practice. Patients discharging from hospitals are often provided with a limited supply of buprenorphine, generally less than two weeks’ worth. By contrast, the regulatory restrictions surrounding methadone that require engagement within an opioid treatment program and that prevent hospitals from dispensing more than three days’ of methadone upon discharge may bias outcome if a significant proportion of TAU participants receive methadone management. Our outcomes analyses will adjust for type of MOUD as a potential confounder, but the trial is not powered to adequately compare each individual MOUD to XR-BUP. Further, as the study progresses, the sites are noting an increasing number of patients who specifically request methadone and do not wish to be on any formulation of buprenorphine. This may be due to perceived fears surrounding buprenorphine and precipitated withdrawal in the era of fentanyl [44], or perceptions of better effect of methadone [45]. For patients who choose methadone-only or who may otherwise decline XR-BUP, we will need strategies beyond those tested in this study to promote post-hospital MOUD engagement.

As hospitals are increasingly addressing OUD, some are already providing XR-BUP as part of the discharge transition process [46]. Currently, in-hospital administration of XR-BUP is not a covered medical benefit, and hospitals are absorbing the cost (>$2000 for combined medication cost and injection procedure fee). Cost effectiveness data are needed to help inform hospitals when choosing to add XR-BUP to inpatient formularies. Positive results from this study could motivate payors to add XR-BUP as a reimbursable hospital medication. Finally, most hospitals do not provide adequate MOUD coverage and efforts, including those of a companion study (CTN-0098B) [47], are needed to facilitate implementation of existing effective interventions.

## Data Availability

No datasets were generated or analysed during the current study.

## References

[1] Bedi P, Rai MP, Bumrah K, Singh VK, Arora TK, Singh T. Pattern and burden of opioid-related hospitalizations in the USA from 2016 to 2018. Br J Clin Pharmacol. 2021;87(11):4366–74. 10.1111/bcp.14857. 33856070 10.1111/bcp.14857

[2] Ronan MV, Herzig SJ. Hospitalizations related to opioid Abuse/Dependence and Associated Serious infections increased sharply, 2002-12. Health Aff (Millwood) May 01. 2016;35(5):832–7. 10.1377/hlthaff.2015.1424. 10.1377/hlthaff.2015.1424 PMC5240777 27140989

[3] Wurcel AG, Anderson JE, Chui KK, et al. Increasing infectious endocarditis admissions among Young people who inject drugs. Open Forum Infect Dis Sep. 2016;3(3):ofw157. 10.1093/ofid/ofw157. 10.1093/ofid/ofw157 PMC5084714 27800528

[4] Njoroge LW, Al-Kindi SG, Koromia GA, ElAmm CA, Oliveira GH. Changes in the Association of Rising Infective Endocarditis With Mortality in People Who Inject Drugs. *JAMA Cardiol*. Aug 1. 2018;3(8):779–780. 10.1001/jamacardio.2018.1602 10.1001/jamacardio.2018.1602 PMC6143071 29926083

[5] King C, Cook R, Korthuis PT, Morris CD, Englander H. Causes of death in the 12 months after hospital discharge among patients with opioid Use Disorder. J Addict Med. 2022;16(4):466–9. 10.1097/adm.0000000000000915. 34510087 10.1097/ADM.0000000000000915 PMC8907339

[6] Priest KC, Lovejoy TI, Englander H, Shull S, McCarty D. Opioid agonist therapy during hospitalization within the Veterans Health Administration: a pragmatic retrospective cohort analysis. J Gen Intern Med. Aug 2020;35(8):2365–74. 10.1007/s11606-020-05815-0. 10.1007/s11606-020-05815-0 PMC7403377 32291723

[7] Santo T, Jr, Clark B, Hickman M, et al. Association of Opioid Agonist Treatment with all-cause mortality and specific causes of death among people with opioid dependence: a systematic review and Meta-analysis. JAMA Psychiatry. 2021. 10.1001/jamapsychiatry.2021.0976. 34076676 10.1001/jamapsychiatry.2021.0976 PMC8173472

[8] MacArthur G J, Minozzi S, Martin N, Vickerman p, Deren s, Bruneau j, et al. Opiate substitution treatment and HIV transmission in people who inject drugs: systematic review and meta-analysis BMJ 2012;345:e5945. 10.1136/bmj.e5945. 10.1136/bmj.e5945 PMC3489107 23038795

[9] Platt L, Minozzi S, Reed J, et al. Needle and syringe programmes and opioid substitution therapy for preventing HCV transmission among people who inject drugs: findings from a Cochrane Review and meta-analysis. Addict Mar. 2018;113(3):545–63. 10.1111/add.14012. 10.1111/add.14012 PMC5836947 28891267

[10] Nosyk B, Bray JW, Wittenberg E, et al. Short term health-related quality of life improvement during opioid agonist treatment. Drug Alcohol Depend Dec. 2015;1:157:121–8. 10.1016/j.drugalcdep.2015.10.009. 10.1016/j.drugalcdep.2015.10.009 PMC4778423 26511766

[11] Larochelle MR, Bernstein R, Bernson D, et al. Touchpoints - opportunities to predict and prevent opioid overdose: a cohort study. Drug Alcohol Depend Nov. 2019;1:204:107537. 10.1016/j.drugalcdep.2019.06.039. 10.1016/j.drugalcdep.2019.06.039 PMC7020606 31521956

[12] Englander H, Davis CS. Hospital standards of Care for people with Substance Use Disorder. N Engl J Med. 2022;387(8):672–5. 10.1056/NEJMp2204687. 35984354 10.1056/NEJMp2204687

[13] Thakrar AP, Lowenstein M, Greysen SR, Delgado MK. Trends in before medically advised discharges for patients with opioid Use Disorder, 2016–2020. JAMA. 2023;330(23):2302–4. 10.1001/jama.2023.21288. 38048121 10.1001/jama.2023.21288 PMC10696509

[14] Zhu H, Wu L-T. Discharge against medical advice from hospitalizations for substance use disorders: The potential impact of the Affordable Care Act. *Drug and Alcohol Dependence*. 2019/04/01/ 2019;197:115–119. doi:10.1016/j.drugalcdep.2018.12.032 10.1016/j.drugalcdep.2018.12.032 PMC6508559 30802735

[15] Marks LR, Munigala S, Warren DK, et al. A comparison of medication for opioid use disorder treatment strategies for persons who inject drugs with invasive bacterial and fungal infections. J Infect Dis Sep. 2020;2(Suppl 5):S513–20. 10.1093/infdis/jiz516. 10.1093/infdis/jiz516 PMC7566615 32877547

[16] Merchant E, Burke D, Shaw L, et al. Hospitalization outcomes of people who use drugs: one size does not fit all. J Subst Abuse Treat May. 2020;112:23–8. 10.1016/j.jsat.2020.01.010. 10.1016/j.jsat.2020.01.010 PMC7089869 32199542

[17] Bahji A, Brothers TD, Mauer-Vakil D, et al. The Effectiveness of Inpatient Addiction Consult Services: a systematic review and narrative synthesis. Can J Addict. 2023;14(2):9–19. 10.1097/cxa.0000000000000173.

[18] Gryczynski J, Nordeck CD, Welsh C, Mitchell SG, O’Grady KE, Schwartz RP. Preventing hospital readmission for patients with Comorbid Substance Use Disorder: a Randomized Trial. Ann Intern Med. Jul 2021;174(7):899–909. 10.7326/m20-5475. 10.7326/M20-5475 PMC13412153 33819055

[19] Marcovitz D, Dear ML, Donald R, et al. Effect of a Co-located Bridging Recovery Initiative on Hospital length of stay among patients with opioid use disorder: the BRIDGE Randomized Clinical Trial. JAMA Netw Open. 2024;7(2):e2356430–2356430. 10.1001/jamanetworkopen.2023.56430. 38411964 10.1001/jamanetworkopen.2023.56430 PMC10900965

[20] Englander H, Dobbertin K, Lind BK, et al. Inpatient Addiction Medicine Consultation and Post-hospital Substance Use Disorder Treatment Engagement: a propensity-matched analysis. J Gen Intern Med Aug. 2019;13. 10.1007/s11606-019-05251-9. 10.1007/s11606-019-05251-9 PMC6854181 31410816

[21] Krawczyk N, Rivera BD, Chang JE, Lindenfeld Z, Franz B. Initiatives to Support the Transition of Patients With Substance Use Disorders From Acute Care to Community-based Services Among a National Sample of Nonprofit Hospitals. *Journal of addiction medicine*. Mar-Apr 01. 2024;18(2):115–121. 10.1097/adm.0000000000001250 10.1097/ADM.0000000000001250 PMC10939963 38015653

[22] Trowbridge P, Weinstein ZM, Kerensky T, et al. Addiction consultation services - linking hospitalized patients to outpatient addiction treatment. J Subst Abuse Treat Aug. 2017;79:1–5. 10.1016/j.jsat.2017.05.007. 10.1016/j.jsat.2017.05.007 PMC6035788 28673521

[23] Roy PJ, Price R, Choi S, et al. Shorter outpatient wait-times for buprenorphine are associated with linkage to care post-hospital discharge. Drug Alcohol Depend Jul. 2021;1:224:108703. 10.1016/j.drugalcdep.2021.108703. 10.1016/j.drugalcdep.2021.108703 PMC8180499 33964730

[24] Marsden J, Kelleher M, Gilvarry E et al. Superiority and cost-effectiveness of monthly extended-release buprenorphine versus daily standard of care medication: a pragmatic, parallel-group, open-label, multicentre, randomised, controlled, phase 3 trial. eClinicalMedicine. 2023/12/01/ 2023;66:102311. 10.1016/j.eclinm.2023.102311 10.1016/j.eclinm.2023.102311 PMC10692661 38045803

[25] Lofwall MR, Nunes E, Bailey G et al. A phase III outpatient randomized, double-blind, double-dummy controlled trial evaluating efficacy of CAM2038 (weekly and monthly buprenorphine FluidCrystal injection depot) for opioid use disorder. Presented at: college on problems of drug dependence: 79th Annual Scientific Meeting; June 19, 2017; Montreal, PQ. Session Oral communications 8. 2017.

[26] National Committee on Quality Assurance. Initiation and engagement of alcohol and other drug dependence treatment: The HEDIS Measure. 10/16/2018, 2018. https://www.ncqa.org/hedis/measures/initiation-and-engagement-of-alcohol-and-other-drug-abuse-or-dependence-treatment/

[27] National Committee on Quality Assurance. NCQA updates quality measures for HEDIS2018 technical specifications update. 2017. April 12, 2019, 2019. https://www.ncqa.org/news/ncqa-updates-quality-measures-for-hedis-2018-technical-specifications-update/

[28] Harris AH, Humphreys K, Bowe T, Tiet Q, Finney JW. Does meeting the HEDIS substance abuse treatment engagement criterion predict patient outcomes? J Behav Health Serv Res Jan. 2010;37(1):25–39. 10.1007/s11414-008-9142-2. 10.1007/s11414-008-9142-2 18770044

[29] Williams AR, Mauro CM, Feng T, et al. Performance measurement for opioid Use Disorder Medication Treatment and Care Retention. Am J Psychiatry Jun. 2023;1(6):454–7. 10.1176/appi.ajp.20220456. 10.1176/appi.ajp.20220456 PMC10130230 36285405

[30] Maiese DR, Hendershot TP, Strader LC, et al. PhenX-Establishing a Consensus process to Select Common measures for Collaborative Research. RTI Press Methods Report Series; 2013. 31145562

[31] Robinson SM, Sobell LC, Sobell MB, Leo GI. Reliability of the Timeline Followback for cocaine, cannabis, and cigarette use. Psychol Addict Behav Mar. 2014;28(1):154–62. 10.1037/a0030992. 10.1037/a0030992 23276315

[32] Sobell LC, Sobell MB, Leo GI, Cancilla A. Reliability of a timeline method: assessing normal drinkers’ reports of recent drinking and a comparative evaluation across several populations. Br J Addict Apr. 1988;83(4):393–402. 10.1111/j.1360-0443.1988.tb00485.x 3395719

[33] Li P, Kim MM, Doshi JA. Comparison of the performance of the CMS Hierarchical Condition Category (CMS-HCC) risk adjuster with the charlson and elixhauser comorbidity measures in predicting mortality. Journal article. BMC Health Serv Res August. 2010;20(1):245. 10.1186/1472-6963-10-245. 10.1186/1472-6963-10-245 PMC2936901 20727154

[34] Wesson DR, Ling W. The Clinical Opiate Withdrawal Scale (COWS). *J Psychoactive Drugs*. 2003;35(2):253–259. NOT IN FILE. 10.1080/02791072.2003.10400007 12924748

[35] American Psychiatric A. Diagnostic and statistical manual of mental disorders (DSM-5) 5th. Arlington, VA: American Psychiatric Publishing; 2013.

[36] Cacciola JS, Alterman AI, Lynch KG, Martin JM, Beauchamp ML, McLellan AT. Initial reliability and validity studies of the revised treatment services review (TSR-6). Drug and Alcohol Dependence. 2008;92(1):37–47. 10.1016/j.drugalcdep.2007.06.004 17644275

[37] Krebs EE, Lorenz KA, Bair MJ, Damush TM, Wu J, Sutherland JM, et al. Development and initial validation of the PEG, a three-item scale assessing pain intensity and interference. J Gen Intern Med. 2009;24(6):733–8. 10.1007/s11606-009-0981-1 PMC2686775 19418100

[38] Kroenke K, Spitzer RL, Williams JB. The PHQ-9: validity of abrief depression severity measure. J Gen Intern Med. 2001;16(9):606–13. 10.1046/j.1525-1497.2001.016009606.x PMC1495268 11556941

[39] Prins A, Bovin MJ, Smolenski DJ, Marx BP, Kimerling R,Jenkins-Guarnieri MA, et al. The primary care PTSD screen for DSM-5(PC-PTSD-5): Development and evaluation within a veteran primary care sample. JGen Intern Med. 2016;31(10):1206–11. 10.1007/s11606-016-3703-5 PMC5023594 27170304

[40] Skevington SM, Lotfy M, O’Connell KA, Group W. The World Health Organization’s WHOQOL-BREF quality of life assessment: psychometric properties and results of the international field trial. A report from the WHOQOL group. Qual Life Res. 2004;13(2):299–310. 10.1023/B:QURE.0000018486.91360.00 15085902

[41] Cacciola JS, Alterman AI, McLellan AT, Lin Y-T, Lynch KG. Initial evidence for the reliability and validity of a “Lite” version of the addiction severity index. Drug and Alcohol Dependence. 2007;87(2):297–302. 10.1016/j.drugalcdep.2006.09.002 17045423

[42] Center for Medicare & Medicaid Services. Hospital Outpatient Prospective Payment System. Accessed May 3. 2019, 2019. https://www.cms.gov/Research-Statistics-Data-and-Systems/Files-for-Order/LimitedDataSets/HospitalOPPS.html

[43] Wittenberg E, Bray JW, Aden B, Gebremariam A, Nosyk B, Schackman BR. Measuring benefits of opioid misuse treatment for economic evaluation: health-related quality of life of opioid-dependent individuals and their spouses as assessed by a sample of the US population. Addict Apr. 2016;111(4):675–84. 10.1111/add.13219. 10.1111/add.13219 PMC5034732 26498740

[44] Spadaro A, Sarker A, Hogg-Bremer W et al. Reddit discussions about buprenorphine associated precipitated withdrawal in the era of fentanyl. *Clinical Toxicology*. 2022/06/03 2022;60(6):694–701. 10.1080/15563650.2022.2032730 10.1080/15563650.2022.2032730 PMC10457147 35119337

[45] Varshneya NB, Thakrar AP, Hobelmann JG, Dunn KE, Huhn AS. Evidence of buprenorphine-precipitated withdrawal in persons who use Fentanyl. J Addict Med. 9000. 10.1097/adm.0000000000000922. 10.1097/ADM.0000000000000922 PMC9124721 34816821

[46] Seval N, Nunez J, Roth P, et al. Inpatient low-dose transitions from full agonist opioids including Methadone onto Long-acting Depot Buprenorphine: Case Series from a Multicenter Clinical Trial. J Addict Med. 2023;17(4):e232–9. 10.1097/adm.0000000000001136. 37579095 10.1097/ADM.0000000000001136 PMC10368784

[47] Bart G, Korthuis PT, Donohue JM et al. Exemplar Hospital initiation trial to Enhance Treatment Engagement (EXHIT ENTRE): protocol for CTN-0098B a randomized implementation study to support hospitals in caring for patients with opioid use disorder. *Addiction Science & Clinical Practice*. 2024/04/11 2024;19(1):29. 10.1186/s13722-024-00455-9 10.1186/s13722-024-00455-9 PMC11007900 38600571

